# Imaging of Bone Marrow Involvement in Lymphoma: State of the Art and Future Directions

**DOI:** 10.1100/tsw.2011.40

**Published:** 2011-02-14

**Authors:** Thomas C. Kwee, John M. H. de Klerk, Rutger A. J. Nievelstein

**Affiliations:** ^1^ Department of Radiology, University Medical Center Utrecht, Utrecht, The Netherlands; ^2^ Department of Nuclear Medicine, Meander Medical Center, Amersfoort, The Netherlands

**Keywords:** lymphoma, bone marrow, imaging, MRI, PET, CT

## Abstract

Accurate detection of bone marrow involvement in patients with lymphoma is of crucial importance because of the prognostic and therapeutic consequences. Bone marrow trephine biopsy (BMB) is currently regarded as the method of choice for the evaluation of the bone marrow in lymphoma, but it is invasive, has a risk of complications, and lacks sufficient sensitivity due to the possibility of sampling errors. Bone marrow imaging, if accurate, may (partially) replace BMBand/or may improve the sensitivity of BMB by guiding the biopsy to the location that appears to be involved by lymphoma at imaging. In this scientific communication, general concepts of bone marrow imaging, state-of-the-art imaging modalities, and future imaging strategies for the assessment of the bone marrow in lymphoma will be reviewed and discussed.

## INTRODUCTION

The lymphomas, broadly divided into Hodgkin and non-Hodgkin lymphomas, comprise approximately 5.0% of all cancers and account for approximately 3.7% of all cancer deaths in the U.S.[1]. Accurate staging is of crucial importance in lymphoma because it determines prognosis and treatment planning[2,3]. Of particular importance is the detection or exclusion of bone marrow involvement, which, if present, by definition indicates the highest stage (stage IV) according to the Ann Arbor staging system[2,3]. The currently used method for the diagnosis of bone marrow involvement is unilateral or bilateral blind bone marrow trephine biopsy (BMB) of the iliac crest. However, BMB is an invasive and painful procedure, and has a small but non-negligible risk of complications[4,5]. An annual survey among 120 hospitals in the U.K. in 2004 documented 15 adverse events (0.07%) among 20,323 BMBs[4]. Hemorrhage was both the most common and the most serious adverse event; it occurred in nine patients. Other patients suffered persistent pain (n=3), collapse relating to previously undiagnosed severe aortic stenosis (n=1), suspected anaphylactic reaction (n=1), and fracture at the site of the biopsy in a patient with concomitant osteoporosis leading to a 2-week hospitalization (n=1)[4]. Besides being invasive, it is important to realize that BMB assesses only a very small portion of the entire bone marrow.

Consequently, although the method has a high specificity, focal bone marrow involvement can be missed by BMB. For example, previous studies showed that in patients with unilaterally proven bone marrow infiltration, contralateral BMB of the iliac crest was negative in 10–60%[6,7,8,9]. It has also been reported that in 33% of paired ipsilateral BMBs of the iliac crest, only one specimen was positive for bone marrow infiltration[8].Because of its disadvantages, it has been suggested that BMB may be omitted in patients with low risk of bone marrow involvement[10] and that it should be performed only in those patients in whom the results will contribute significantly to management decisions or serve as a useful prognostic indicator[5]. Another way to reduce the number of BMBs may be achieved by means of imaging. That is, imaging may be able to detect or exclude the presence of bone marrow involvement, thereby eliminating the need to perform BMB. Alternatively, if imaging indicates the possible presence of bone marrow involvement and histological confirmation is desired, imaging findings may guide and improve the sensitivity of BMB. In this scientific communication, general concepts of bone marrow imaging, state-of-the-art imaging modalities, and future imaging strategies for the assessment of the bone marrow in lymphoma will be reviewed and discussed.

## GENERAL CONCEPTS OF BONE MARROW IMAGING

Cancer cells are lodged in the bone marrow as the initial site for skeletal metastasis by means of hematogeneous spread[11,12]. This has important consequences for imaging in that an imaging modality should be able to visualize the bone marrow in order to detect (early) bone metastatic disease. Another important concept is that bone marrow metastases are most frequently (>90%) localized in the hematopoietic (red) marrow because of its richer blood supply compared to fatty (yellow) marrow. Consequently, the localization of bone marrow metastases is dependent on the distribution of the red marrow. At birth, visually all marrow is of the red type. Conversion of red to yellow marrow begins in the postnatal period, first in the extremities, progressing from the peripheral towards the axial skeleton and from diaphysis to the metaphysis of individual long bones. By the age of 25 years, marrow conversion is usually complete, and red marrow is predominantly seen in the axial skeleton and in the proximal part of the appendicular skeleton. Consequently, in adults, the most common sites for bone marrow metastases are the vertebrae (69%), pelvis (41%), proximal femoral metaphyses (25%), and skull (14%)[11,12]. Therefore, if imaging is employed to screen for lymphomatous lesions in the bone marrow, at least the volume from the skull to the proximal femoral metaphyses should be included in the field of view (FOV).

Imaging modalities that are used in routine clinical practice for the evaluation of the skeletal system include conventional radiography, computed tomography (CT), bone scintigraphy, positron emission tomography (PET), and magnetic resonance imaging (MRI). Conventional radiography is relatively inexpensive, widely available, and of great value in the assessment of cortical and trabecular bone. However, it yields projection images only, is not suitable to assess the bone marrow, and, moreover, a change of 30–50% in mineral density is needed before a bone lesion becomes visible[13]. CT is very suitable to visualize cortical and trabecular bone, is more sensitive than conventional radiography[14,15], but is not an appropriate method for bone marrow assessment[16]. Bone scintigraphy is regarded as a useful screening method for bone metastatic disease, but misses metastases confined to the bone marrow and metastases that do not induce any osteoblastic reaction[17,18].Unlike conventional radiography, CT, and bone scintigraphy, PET using the radiotracer $^{18}$F-fluoro-2-deoxy-D-glucose (FDG) and MRI have the great advantage of being capable of visualizing the bone marrow and detecting bone marrow metastases at an early stage, before bone remodeling has occurred. Another important advantage of PET and MRI is their whole-body imaging capability, potentially allowing for complete nodal and extranodal staging, including the bone marrow (Fig. 1). The utility of FDG-PET and MRI in the assessment of bone marrow involvement in lymphoma will be described in the following two sections.

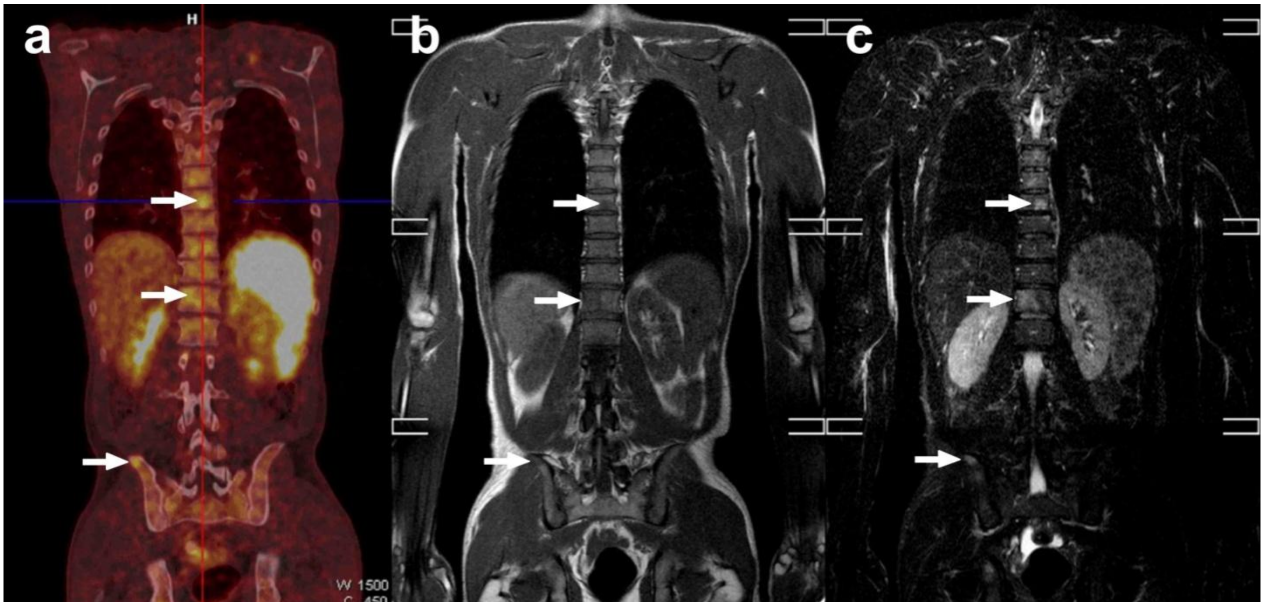

**FIGURE 1.** Coronal FDG-PET/CT (a), coronal T1-weighted MRI (b), and coronal STIR (short-inversion time-inversion recovery)whole-body MRI (c) in a 45-year-old male with nodular sclerosing Hodgkin lymphoma. Both FDG-PET/CT and MRI show vertebral and iliac bone marrow lesions (arrows). Blind BMB of the right posterior iliac crest confirmed lymphomatous bone marrow involvement. Both imaging modalities also detected extensive supra- and infra-diaphragmatic lymphadenopathy and splenic involvement (not shown).

## FDG-PET

The glucose analogue FDG is currently the most frequently used PET radiotracer in clinical practice. The rationale for the use of FDG for PET imaging in oncology is the fact that the vast majority of malignant cancer phenotypes exhibit an increased glycolysis under aerobic conditions (i.e., the Warburg effect)[19]. This is also true for most lymphoma subtypes, with the exception of extranodal marginal zone lymphoma and small lymphocytic lymphoma[20]. Imaging of lymphoma with FDG-PET was first described in 1987[21], and numerous subsequent studies have validated and confirmed the utility of FDG-PET for staging lymphoma[22]. Most FDG-PET studies are currently performed as part of a combined PET/CT examination. The CT component of a combined PET/CT system offers well-recognized advantages, among which are more accurate localization of sites with FDG uptake and its use for attenuation correction of PET emission data, which provides low-noise attenuation correction factors, eliminates bias from emission contamination of post injection transmission scans, and allows for whole-body FDG-PET scanning in 20 min or less[23]. FDG-PET is playing an increasingly important role in the evaluation of patients with lymphoma, both for initial staging (at diagnosis) and restaging (after onset or completion of therapy)[22]. FDG-PET directly images (tumor) cells based on their metabolic activity, making it a suitable tool for the evaluation of the bone marrow in patients with lymphoma (Fig. 1)[24,25].

Several studies have investigated this application of FDG-PET, but most of them were limited by relatively small sample sizes[25]. To overcome these limitations, Pakos et al.[25] performed a meta-analysis of 13 studies comprising a total of 587 patients to assess the diagnostic performance of FDG-PET in evaluating the bone marrow in patients with primary lymphoma or recurrent lymphoma after complete remission. In this meta-analysis, the independent random-effects weighted estimates of sensitivity and specificity of FDG-PET against BMB were 51% (95% confidence interval[CI], 38–64%) and 91% (95% CI, 85–95%), respectively. Based on these findings, Pakos et al.[25] concluded that FDG-PET cannot yet be recommended for replacing BMB routinely in the staging of lymphoma because many cases of bone marrow involvement would be missed. Interestingly, however, six of 12 patients with positive FDG-PET and negative initial BMB were found to have bone marrow involvement when BMB was performed at the sites with positive imaging signals. Thus, FDG-PET could complement BMB and could occasionally identify additional cases of focal bone marrow involvement that would be missed by BMB alone. Another interesting finding of this meta-analysis is that sensitivity of FDG-PET was higher in patients with Hodgkin lymphoma and in high-grade non-Hodgkin lymphomas than in patients with less aggressive histologic subtypes, such as follicular, mantle cell, (extranodal) marginal zone, and small lymphocytic lymphomas[25]. This can be explained by the fact that Hodgkin lymphoma and high-grade non-Hodgkin lymphomas are known to exhibit higher FDG uptake than low-grade non-Hodgkin lymphomas[20]. The findings of this meta-analysis have been confirmed by subsequent studies using state-of-the-art PET/CT systems[26,27,28,29].Thus, in summary, FDG-PET and BMB should be regarded as complementary, and the diagnostic yield of the former is higher in high-grade lymphomas than in low-grade lymphomas. Since FDG-PET/CT is being used increasingly as the first-line imaging modality for staging lymphoma, a strategy in which FDG-PET/CT is performed first, followed by a FDG-PET/CT–guided BMB (if FDG-PET/CT is positive) or a blind BMB (if FDG-PET/CT is negative) may well become the method of choice for assessing the bone marrow in lymphoma in the near future. However, further investigations are required to show the utility of this strategy.

Future advances in PET technology that provide a higher signal-to-noise ratio (SNR)and a higher spatial resolution are likely to increase the diagnostic performance of FDG-PET. In this context, the reintroduction of time-of-flight(TOF) PET may play an important role[23,30]. PET systems with TOF capabilities are currently commercially available for clinical practice[23,30]. In TOF PET, the actual time difference in the arrival of the two annihilation photons at the detectors is recorded. The TOF information is incorporated directly into the reconstruction algorithm, permitting some combination of faster scanning, improved SNR, or improved spatial resolution[23,30]; the latter two may improve the detection of small bone marrow lesions, although future clinical studies are still needed to prove this assumption. It should also be investigated whether the detection of additional (small) bone marrow lesions has any relevant clinical consequences, particularly with regard to the prediction of progression-free or overall survival. This is not only applicable to FDG-PET, but also to other diagnostic (imaging) tests. Another possibility to improve the diagnostic yield of FDG-PET may be through performance of delayed PET imaging (i.e., 3 or 4 h after FDG administration). The rationale for delayed imaging is based on the fact that several tumors exhibit a maximum FDG uptake well beyond 60 min after FDG administration, while surrounding normal tissues and benign pathologies show a decline in FDG uptake with time[31,32,33,34]. This phenomenon occurs because most types of malignant cells have significantly increased ratios of hexokinase to glucose-6-phosphatase activities, which allows FDG to accumulate in much higher levels over time than in normal cells and benign pathologies. As such, contrast between lymphomatous bone marrow and normal red bone marrow can be increased, while FDG uptake of benign bone pathologies can be decreased at delayed PET imaging. This, in turn, would increase both sensitivity and specificity of FDG-PET. Future studies are required to investigate the utility of delayed FDG-PET imaging in the detection of bone marrow involvement in lymphoma.

## MRI

MRI allows direct visualization of all bone marrow components at a good spatial resolution[35,36]. Most MRI systems currently operate at a field strength of 1.5T, but it is expected that more and more MRI examinations will be performed at 3.0T, which provides higher SNR and potentially improves lesion detectability[37]. On the other hand, there is no consensus yet which (combination) of MRI sequences provide the highest diagnostic yield in assessing the bone marrow in lymphoma while being time efficient. Table 1 summarizes the main characteristics of several MRI sequences that can be used for the evaluation of the bone marrow in patients with lymphoma. Sequences that can be regarded as indispensable for the evaluation of the bone marrow are T1-weighted and fat-saturated T2-weighted, or

**TABLE 1**
**Main Characteristics of MRI Sequences that can be Used for the Evaluation of the Bone Marrow in Patients with Lymphoma**

| Sequence | Principle | Appearance of Lymphomatous Bone Marrow Lesions |
|---|---|---|
| T1-weighted imaging | Provides tissue contrast that is mainly based on differences in T1 relaxation times | Relatively low signal intensity |
| Fat-saturated T2-weighted imaging | Provides tissue contrast that is mainly based on differences in T2 relaxation times and suppresses signal from surrounding fatty tissues | Relatively high signal intensity |
| Short-inversion time-inversion recovery (STIR) imaging | Sensitive to prolongation of both T1 and T2 relaxation times and suppresses signal from surrounding fatty tissues | Relatively high signal intensity |
| Diffusion-weighted imaging (DWI) | Provides tissue contrast that is mainly based on differences in diffusivity and T2 relaxation times, and suppresses signal from surrounding fatty tissues | Relatively high signal intensity |
| Gadolinium-enhanced T1-weighted imaging (images should either be acquired with fat suppression or both before and after administration of gadolinium) | Gadolinium is taken up by lymphomatous bone marrow lesions, as a result of which their T1 relaxation times decrease, which, in turn, improves their conspicuity | Relatively high signal intensity |
| Ultra small particles of iron oxide (USPIO)–enhanced fat-saturated T2-weighted, STIR, or DWI | USPIOs are taken up by both the red and yellow marrow, as a result of which their T1, T2, and T2* relaxation times decrease, which, in turn, improves conspicuity of lymphomatous bone marrow lesions that do not take up USPIOs | Relatively high signal intensity |

short-inversion time-inversion recovery (STIR) sequences[38,39]. Lymphomatous bone marrow lesions typically have longer T1 and T2 relaxation times than normal yellow and red marrow (i.e., low signal intensity on T1-weighted images and high signal intensity on fat-saturated T2-weighted or STIR images), because they contain larger amounts of water and lesser amounts of fat (Fig. 1). Gadolinium-enhanced images can improve the detection of bone marrow metastases; normal marrow usually shows no visible contrast enhancement, whereas bone marrow metastases often exhibit strong signal increase. On the other hand, the addition of gadolinium-enhanced sequences prolongs scan time and may cause contrast media-induced side effects. Furthermore, STIR imaging has been reported to be at least equal to gadolinium-enhanced MRI for the detection of bone marrow lesions[40,41]. For this reason, it can be argued that gadolinium-enhanced sequences may be omitted from the MRI examination for the detection of lymphomatous bone marrow lesions. Another sequence that may be useful for the evaluation of the bone marrow is diffusion-weighted imaging (DWI)[42,43]. DWI is basically a fat-suppressed T2-weighted sequence that is sensitive to the random (Brownian) motion of water molecules. Yellow marrow is suppressed at DWI thanks to the use of a fat-suppression prepulse, while red marrow demonstrates higher signal intensity because of its relatively high cellularity and water content. Because many malignant tumors, including lymphomatous bone marrow lesions, have both a prolonged T2 relaxation time and an impeded diffusion, they usually exhibit high signal intensity at DWI[42,43]. The advantage of DWI over conventional (T1- and T2-weighted) sequences is expected to lie in its good lesion-to-background contrast. However, the additional value of DWI over conventional sequences in the detection of lymphomatous bone marrow lesions has not yet been proven[39,44].In general, MRI can be regarded as a very sensitive technique for the detection of bone marrow lesions. However, its specificity may be suboptimal[45]. In particular, the discrimination between reconverted or red marrow (islands), benign bone marrow pathologies (such as inflammatory infiltrates), and lymphomatous bone marrow lesions may sometimes be difficult due to overlap in signal intensities[35,36].

The potential utility of MRI for the detection of bone marrow involvement in lymphoma had already been investigated in the late 1980s[46] and several subsequent studies have been performed on this issue[47]. A recent systematic review including 11 studies reported that sensitivity of MRI for the detection of bone marrow involvement ranged from 50 to 100%, with a median of 100%[47].However, besides several methodological shortcomings, most of these studies had relatively small sample sizes, used different sequences, and only the most recent studies used state-of-the-art MRI hardware and sequences[47]. Furthermore, the majority of previous studies applied MRI protocols that examine only selected body regions (mostly lumbar spine, pelvis, and proximal femurs)[47]. Major disadvantages of these MRI protocols are that bone marrow involvement outside the image volume is missed and they do not allow complete lymphoma staging because of the absence of full body coverage. However, thanks to technological advances, including the development of high-performance magnetic field gradients, parallel imaging, faster MRI sequences, and new coil and table concepts, it is currently possible to use whole-body MRI as a “one-stop-shop” staging procedure in lymphoma, making it a potential alternative to FDG-PET/CT[48,49]; in this setting, MRI may become more time efficient and cost effective than as an add-on tool for the assessment of the bone marrow only. However, it should be noted that whole-body MRI is generally not comparable to MRI protocols of selected body regions, since the former allows less time to acquire different MRI sequences and imaging planes, and generally employs a greater slice thickness and lower spatial resolution. Three studies[44,50,51] reported the value of whole-body MRI for bone marrow assessment in lymphoma compared to BMB. Brennan et al.[50] performed axial and coronal STIR whole-body MRI and sagittal T1-weighted MRI of the spine in 23 patients with lymphoma. Seventeen patients were in clinical remission and six were being actively treated for disease. BMB results were available in 18 patients. MRI correctly predicted marrow invasion in two patients, and also correctly predicted normal or non invaded marrow in 16 patients[50]. In another study by Ribrag et al.[51], coronal T1-weighted and STIR whole-body MRI were performed in 43 patients with newly diagnosed malignant lymphoma. In nine patients, whole-body MRI showed focal bone abnormalities, of which two were confirmed by BMB. The other 34 patients had no bone marrow involvement at whole-body MRI or BMB[51]. A limitation of the studies by Brennan et al.[50] and Ribrag et al.[51] is that a very low number of patients with histologically proven bone marrow involvement were included (n=2 in both studies). In a larger study by Kwee et al.[44], 48 patients, of whom 12 had a positive BMB, underwent coronal whole-body MRI (T1-weighted and STIR [n=48] and DWI[n=44]). Whole-body MRI without DWI and whole-body MRI with DWI were positive in only five of 12 and five of 11 patients with a positive BMB, respectively. Based on the results of this study[44], it can be concluded that whole-body MRI is currently not yet sufficiently reliable to replace BMB for bone marrow assessment in lymphoma. Interestingly, however, in eight patients, whole-body MRI (both without and with DWI) was positive, while BMB was negative. However, whether whole-body MRI provided correct upstaging or not in these cases remained unclear because histopathological verification was not possible and follow-up imaging studies were not available[44]. Nevertheless, it is of interest to note that a previous study already reported that lymphoma patients with MRI findings positive for bone marrow involvement, but a negative BMB, have a significantly shorter survival than those without a negative MRI for bone marrow involvement[52]. Future studies are necessary to determine whether whole-body MRI can be complementary to BMB.

There may be several ways to improve the diagnostic performance of MRI in the assessment of the bone marrow in lymphoma. First, as mentioned previously, it may be beneficial to perform MRI at higher field strength since SNR increases linearly with field strength[37]. The higher SNR may facilitate the detection of small lymphomatous bone marrow lesions, although this still has to be proven in clinical studies. Another way may be by means of performing MRI after administration of ultra small particles of iron oxide (USPIOs)[53,54,55,56].Intravenously administered USPIOs are taken up by macrophages in the reticuloendothelial system, predominantly within the lymph nodes, but also in red and yellow marrow[53,54,55,56]. Approximately 1 h after administration of USPIOs, T1,T2, and T2* relaxation times of normal red and yellow marrow are considerably lower than precontrast relaxation times. Consequently, hyperplastic or normal red marrow exhibits low signal intensity on USPIO-enhanced fat-saturated T2-weighted, STIR, and DWI. On the other hand, bone marrow metastases do not take up USPIOs, as a result of which their relaxation times do not decrease. Consequently, bone marrow metastases maintain relatively high signal intensity on USPIO-enhanced fat-saturated T2-weighted, STIR, and DWI[53,54,55,56]. Thus, this approach could improve the differentiation between residual or reconverted red marrow (islands) and lymphomatous bone marrow lesions. In a study including nine patients with non-Hodgkin lymphoma who underwent both noncontrast-enhanced MRI and USPIO-enhanced MRI of the spine, it was shown that contrast between bone marrow metastases and normal bone marrow on STIR images can significantly ($p$ <0.05) be increased after USPIO administration[55]. In another study in 22 patients with non-Hodgkin lymphoma (including nine patients before and 13 patients after conditioning therapy) who underwent both noncontrast-enhanced MRI and USPIO-enhanced MRI of the spine, it was reported that overall lesion detectability was significantly ($p$ =0.002)better on the postcontrast images compared to the precontrast images. In detail, significantly ($p$ =0.006)more lesions <1 cm were depicted on the postcontrast images, but for lesions >1 cm, no significance could be detected ($p$ =0.8)[56]. Unfortunately, the availability of the current generation of USPIOs is very limited. Nevertheless, effectiveness and safety of a new generation of USPIOs (P904; Guerbet Laboratories, Paris, France) are being tested in preclinical studies at the moment. Advantages of this new USPIO agent are its faster blood pharmacokinetics and the early uptake in the reticuloendothelial system. This, in turn, may decrease the necessary time between USPIO administration and actual scanning. It is expected that this new contrast agent will get approval for clinical applications within the next few years, and its application in MRI of the bone marrow has great potential.

## FUTURE CONSIDERATIONS

### FDG-PET/MRI

Both FDG-PET and MRI are able to visualize the bone marrow and to detect bone marrow metastases at an early stage. However, both modalities provide image contrast based on completely different biophysical and biochemical underpinnings. Therefore, the information provided by FDG-PET and MRI can be regarded as complementary, and the combination of both may improve the detection of lymphomatous bone marrow lesions compared to either of the two alone. For example, FDG-PET may provide a better contrast between lymphomatous bone marrow lesions and (residual or reconverted) red bone marrow (islands)/benign bone marrow pathologies because of(large)differences in metabolic activity, whereas MRI may have difficulties in differentiating these entities because of similarities in signal intensity. On the other hand, achievable spatial resolution of MRI is higher than that of PET, as a result of which the latter may be able to detect smaller lymphomatous bone marrow lesions. MRI may also detect lymphomatous bone marrow lesions in patients with low-grade lymphomas that remain unnoticed at FDG-PET because of low or no FDG uptake, commensurate with their less aggressive biology. There are three major ways to technically integrate PET and MRI systems[57]. The first approach is to perform separate imaging in devices placed far from each other; the patient has to get off one and onto the other imaging system during the examination process. However, this method is rather time-consuming and may suffer from a considerable geometrical mismatch between the PET and MRI datasets. The second approach is to perform sequential imaging by systems that are linked by a patient “shuttle”; the patient does not have to get off the examination table of one device to get onto the table of the second device as the transfer is accomplished by the “shuttle”. This method overcomes many of the disadvantages of the first approach, although the CT component of the PET/CT system is still necessary for transmission scanning. The first sequential whole-body PET/MRI systems have recently been installed in several institutions and the oncological applications are currently under clinical evaluation. The third major way is by means of fully integrated systems with technically simultaneous data acquisition; neither patient nor table motion is required when imaging single FOV[57]. True simultaneous scanning minimizes the geometrical mismatch between PET and MRI dataset. However, several technological difficulties have to be solved for designing a fully integrated whole-body PET/MRI system, including the issues of electromagnetic interference between the two systems and MRI-based attenuation correction[57].Last but not least, the cost effectiveness of this new technology should be evaluated.

## Dual-Energy CT

CT (either as a stand-alone imaging modality or as part of a combined PET/CT examination) is an important method that is routinely used for the evaluation of patients with lymphoma. However, the utility of CT in the detection of early metastatic deposits in the bone marrow has been limited so far[16]. This is due to the fact that the depiction of the bone marrow on CT is impeded by the overlying bone. Furthermore, although postprocessing software allows one to remove bone structures from the image, the bone marrow cannot be visualized because the delicate trabecular bone structures surrounding the bone marrow cannot be resolved. However, the recent introduction of dual-energy CT systems may enhance the role of this imaging modality in the evaluation of the bone marrow[58,59].Technologic advances in dual-energy CT systems have been triggered by the introduction of dual-source CT systems that were primarily developed to achieve a higher temporal resolution for cardiac imaging. Unlike conventional CT systems that can utilize only one X-ray energy source and one X-ray detector at one time, dual-energy CT systems can use two different X-ray energy sources (that can be operated at different potentials) with two corresponding X-ray detectors at one time. As such, it is possible to simultaneously acquire two datasets at two different photon energies in a single acquisition. By obtaining CT data at different photon energies and by using various dual-energy postprocessing algorithms that are based on three-material decomposition principles, differences in material composition can be visualized and quantified based on differences in photon absorption. This works especially well in materials with large atomic numbers, such as iodine and calcium, because these materials exhibit relatively large differences in photon absorption at different (low and high)photon energy settings[58,59].Interestingly, a recent feasibility study has indicated that it may be possible to use dual-energy CT for bone marrow assessment[60]. In this study, Pache et al.[60] reported a novel dual-energy CT postprocessing algorithm based on three-material decomposition (bone mineral, yellow marrow, and red marrow), which was termed “virtual noncalcium”. The dual-energy CT virtual noncalcium technique subtracts calcium from trabecular bone, allowing for bone marrow assessment, and has shown promise for the detection of post-traumatic bone bruises of the knee in a small series of 21 patients[60].It is conceivable that this method may also allow for the detection of lymphomatous bone marrow involvement at an earlier stage than conventional single-source CT systems. In this respect, it would be of interest to integrate this new dual-energy CT technology in combined PET/CT systems. However, clinical studies on the use of dual-source CT in the assessment of the bone marrow in patients with cancer, including lymphoma, are still lacking. Despite its promise, dual-energy CT still has several limitations. First, one of the two X-ray tubes has a smaller FOV than the other, which has standard FOV. Moreover, the very peripheral FOV of the former cannot be utilized for dual-energy CT postprocessing. Therefore, in large/wide patients, a part of the bone marrow will not be included in the image volume. Second, datasets obtained at lower photon energy settings inherently have more noise than images acquired at higher photon energy settings. As a result, dual-energy acquisitions are ineffective in very large/wide or obese patients[58,59]. Third, attenuation changes of bone marrow lesions on (postprocessed) dual-energy CT images are more subtle than corresponding signal-intensity alterations on MRI[60].Another limitation of the dual-energy CT approach reported by Pache et al.[60] is the inability of this method to show marrow alterations directly adjacent to cortical bone owing to incomplete masking of the cortex and spatial averaging. Finally, and perhaps most importantly, the virtual noncalcium algorithm introduced by Pache et al.[60] may be less suitable for investigations of bones with a predominantly red marrow composition (note that the red bone marrow is the site where >90% of lymphomatous bone marrow lesions are located) because water (i.e., edema) could be simulated by a certain mixture of red and yellow marrow. Therefore, further technologic developments are necessary before dual-energy CT can become a feasible method for the evaluation of the bone marrow in patients with lymphoma.

## CONCLUSION

FDG-PET and MRI have the advantage of being capable of directly visualizing the bone marrow. Since the bone marrow is the primary location of skeletal metastasis, FDG-PET and MRI can detect bone marrow metastases at an early stage, before bone remodeling has occurred. Furthermore, the whole-body imaging capability of PET and MRI makes them potentially suitable methods for complete nodal and extranodal staging, including the bone marrow. Current evidence indicates that FDG-PET and MRI cannot yet replace BMB, but also suggests that both methods can be complementary to BMB. Technologic developments and new concepts (including delayed imaging for FDG-PET and bone marrow contrast agents for MRI) may improve the diagnostic performance of both methods in the detection of lymphomatous bone marrow lesions. Other promising methods that may improve the evaluation of the bone marrow in lymphoma are combined PET/MRI systems and dual-energy CT. However, future (clinical) studies are needed to optimize and validate these new technologies.

## ACKNOWLEDGMENTS

This work was supported by a ZonMW AGIKO stipend (grant number 92003497) for T.C.K.
